# The prefrontal cortex and (uniquely) human cooperation: a comparative perspective

**DOI:** 10.1038/s41386-021-01092-5

**Published:** 2021-08-19

**Authors:** Yoonseo Zoh, Steve W. C. Chang, Molly J. Crockett

**Affiliations:** Department of Psychology, Yale University, New Haven, USA

**Keywords:** Social neuroscience, Decision

## Abstract

Humans have an exceptional ability to cooperate relative to many other species. We review the neural mechanisms supporting human cooperation, focusing on the prefrontal cortex. One key feature of human social life is the prevalence of cooperative norms that guide social behavior and prescribe punishment for noncompliance. Taking a comparative approach, we consider shared and unique aspects of cooperative behaviors in humans relative to nonhuman primates, as well as divergences in brain structure that might support uniquely human aspects of cooperation. We highlight a medial prefrontal network common to nonhuman primates and humans supporting a foundational process in cooperative decision-making: valuing outcomes for oneself and others. This medial prefrontal network interacts with lateral prefrontal areas that are thought to represent cooperative norms and modulate value representations to guide behavior appropriate to the local social context. Finally, we propose that more recently evolved anterior regions of prefrontal cortex play a role in arbitrating between cooperative norms across social contexts, and suggest how future research might fruitfully examine the neural basis of norm arbitration.

## Introduction

Relative to other species, humans have an exceptional ability to cooperate—we are willing to incur personal costs to benefit others, including strangers, and people who we will never meet again [1–7] (see Glossary). These abilities are thought to arise from complex systems of shared moral intuitions about what is “right” or “good” that are culturally transmitted across space and time [8, 9]. Here, we examine the neurocognitive processes that contribute to uniquely human cooperation, focusing on the prefrontal cortex, which has dramatically expanded over the course of human evolution [10] (see Preuss & Wise, this issue).

To organize our review of the prefrontal cortex and human cooperation, we adopt a comparative approach, considering similarities and differences between humans and nonhuman primates in cooperative behavior and its neural underpinnings. In bridging these bodies of research, we identify gaps in our understanding of the neurobiology of cooperation and suggest directions for future research. A comparative behavioral approach allows us to consider how humans navigate social interactions relative to nonhuman primates, including which aspects of cooperative behavior are unique to humans. Likewise, a comparative neuroscience approach can help identify brain mechanisms that may be unique to humans vs. shared with other nonhuman primate species.

**Fig. 1:**
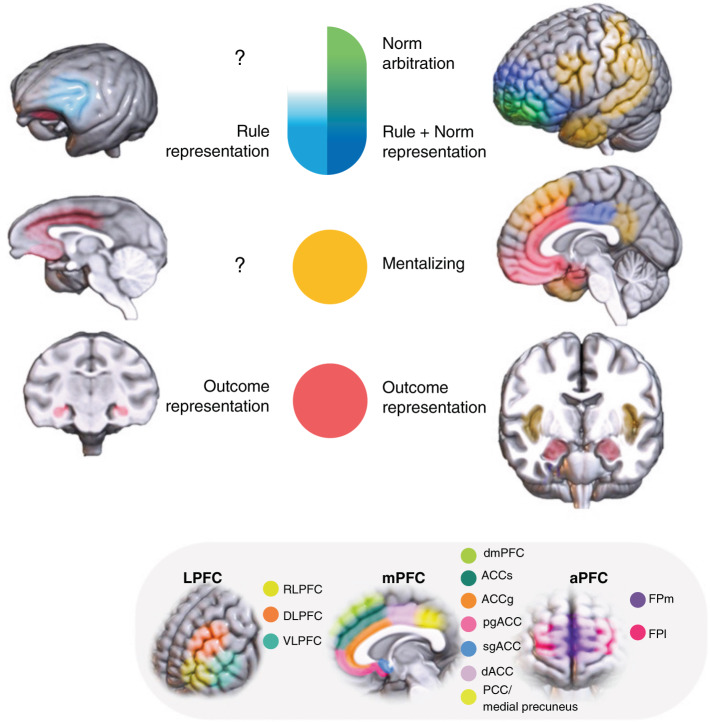
Brain networks for human cooperation and functional homologies in primates. We highlight four brain networks that play complementary but distinct roles in human cooperation, with the functionally corresponding regions in the brain of nonhuman primate depicted where applicable. An outcome representation network encodes motivationally salient outcomes for self and other and encompasses ACC, dmPFC, amygdala, lOFC, and vmPFC. A second network is recruited during mentalizing, a collection of structures including ACC, dmPFC, pSTS, temporal sulcus, temporal pole, TPJ, medial precuneus, and PCC. A third network is activated when a norm is represented and coordinates the outcome values for self and other. This encompasses the regions of dlPFC, dACC, inferior frontal gyrus, and anterior insula. A fourth network is proposed to be engaged in cooperative norm arbitration and encompasses anterior prefrontal regions including FPC and alPFC. We note that some functional correspondences between humans and nonhuman primates have not been adequately explored to make a firm conclusion, which are denoted by question mark. The specific regions of prefrontal areas we focus on are labeled to clearly identify the distinct roles of subregions reported in previous works.

In considering the neural underpinnings of human cooperation, we build on prior hypotheses that the prefrontal cortex, especially its more recently evolved anterior components, supports advanced cognitive functions that are unique to humans [11, 12] (see Preuss & Wise, this issue). We note that human cooperation also draws heavily on brain structures outside the prefrontal cortex, as shown in Fig. 1. Among the brain regions implicated in human cooperation, some areas (e.g., the temporoparietal junction or TPJ) do not show structural correspondences between primates and humans. Also, the lack of systematic and controlled comparative studies poses a challenge in drawing conclusions about functional correspondences of certain brain regions between primates and humans. Our primary focus here is on identifying how prefrontal networks support uniquely human aspects of cooperation while also highlighting functional homologies between human and nonhuman primates whenever applicable.

One critical challenge in describing the neural basis of human cooperation concerns defining the functional boundaries between social cognition, and domain-general cognition. Indeed, whether there are neural mechanisms that are specifically “social” remains a topic of ongoing debate [13, 14]. Here, we adopt a view that there are unlikely to be stark categorical boundaries between so-called “social”, and “non-social” cognition. For instance, there is evidence for overlapping circuits processing social and non-social rewards [15, 16], and that social decision-making in humans draws upon domain-general valuation processes [17–19]. However, there is also an alternative possibility that there exists a population of neurons specialized for social processing in the same neural circuitry. Nevertheless, the spatial resolution of fMRI precludes drawing firm conclusions about whether indeed the very same neurons are engaged in value computation during social and non-social decisions, leaving open the possibility for domain specificity at a higher spatial or temporal resolution. For the purposes of this review, we therefore focus on the neural mechanisms of domain-general cognitive processes that likely play a central role in social cognition, while remaining agnostic about the question of domain specificity at a neuronal level.

In this paper, we will first highlight a few key similarities and differences between nonhuman primates and humans in cooperative behaviors. Next, we will survey the prefrontal networks engaged in human cooperative behavior. We start by examining the role of medial prefrontal and striatal systems in the cognitive foundations of cooperative behavior that are present in both nonhuman primates and humans: valuing outcomes for oneself and others, and predicting others’ behavior. We then turn to research on cooperative decision-making in humans, reviewing evidence that the lateral prefrontal cortex orchestrates the pursuit of cooperative goals by representing cooperative norms and modulating value representations to guide behavior appropriate to the local social context. Finally, we propose that more recently evolved anterior regions of the prefrontal cortex might play a role in arbitrating between cooperative norms across social contexts, and suggest how future research might fruitfully examine the neural basis of norm arbitration.

## Uniquely human cooperation?

Comparative studies of cooperation across species suggest that human cooperation is remarkably sophisticated [20, 21]. Behavioral research on this topic occupies a vast literature (for comprehensive reviews, see [22–25]) and there remains debate about which aspects of cooperation (if any) are unique to humans. Many cooperative behaviors in humans and nonhuman species alike can be explained by kinship [26, 27], cooperative breeding [28, 29], and reciprocity [30–33]. However, these mechanisms cannot fully explain the richness and complexity of human cooperation, which encompasses not just cooperation with kin and the repayment of favors, but also cooperation in the absence of direct reciprocal benefits and a commitment to enforcing cooperative principles even when one is not directly affected by uncooperative behavior. In this section, we identify a few key cognitive processes that may undergird extensive cooperation in humans: self-regulation, metacognition, mentalizing, shared intentionality and norm representation. Our goal here is not to provide an exhaustive review, but rather to highlight a few differences across species to set the stage for our central question: how the prefrontal cortex supports human cooperation.

### Self-regulation and metacognition

A critical feature of human cooperation is a capacity for self-regulation, i.e., adjusting one’s inner states or behaviors according to personal goals, expectations, and standards [34]. Cooperative behavior requires (1) understanding that personal desires often conflict with those of specific others or societal welfare more broadly, (2) regulating those desires in order to behave appropriately, and (3) recognizing when one’s behavior falls short of others’ expectations in order to improve in the future. These abilities draw on the cognitive processes of metacognition and self-regulation, capacities that are substantially more advanced in humans than nonhuman primates.

In humans, there is evidence that cooperative abilities are related to self-regulation abilities, both in economic games and naturalistic settings [35–38]. Notably, self-regulation abilities (as indexed by reduced discounting of delayed rewards) are markedly more advanced in humans than nonhuman primate species, which may be related to cross-species differences in the scale and scope of cooperation [39].

One particular aspect of self-regulation that may be unique to humans is precommitment: the voluntary restriction of access to temptations[40–46]. For example, dieters might avoid purchasing unhealthy foods so as not to be tempted at home, or people looking to save up for a big purchase might lock funds away in accounts with high early withdrawal fees. In a cooperative setting, precommitment can take the form of a social contract, where all parties agree in advance to adhere to a set of mutually agreed-upon rules or expectations. Indeed, both formal and informal social contracts are a central feature of human moral life and may contribute to humans’ extraordinary scope of cooperation over time and space.

Precommitment in humans relies on a metacognitive insight that one’s own self-regulation is likely to fail in the absence of a binding contract [47]. Metacognition is the ability to monitor, assess and orchestrate one’s own cognitive processes and their quality for the guidance of behavior [48–53]. There is evidence for metacognitive abilities in nonhuman primates [54–60], often operationalized as selective information-seeking behavior when more information needs to be collected to make an informed decision, or confidence judgements indexed by different amounts of wagers on the accuracy of one’s performance. While the ability to assess one’s prior experience might be shared between human and nonhuman primates, however, metacognition in humans with the use of language and narrative form implicates far more extensive and complex construction of mental models [61]. The advanced metacognitive ability in humans to introspect upon the effectiveness of one’s performance also involves increased use of strategies and opportunities for improvement in the future [62–64]. The extensive metacognitive ability in humans prompts the reflection of one’s quality as a cooperative partner and further social engagement [65–67].

### Mentalizing and shared intentionality

In addition to monitoring and regulating our own cognitive processes, humans also monitor the cognitive processes of others through mentalizing, and communicate the contents of these thought processes with others [67, 68]. While our nonhuman primate relatives can represent what other agents see and know to make informed predictions about their behavior, there is limited evidence that they can represent others’ beliefs, in particular false beliefs and ignorance [69]. On the other hand, humans develop early in life the ability to infer and understand the dynamic mental states of others that are distinct from one’s own, even when those mental states deviate from reality [70–75]. Humans also spontaneously attribute mental states to others to make sense of others’ behavior as arising from intentional stance [76]. Metacognition and mentalizing enhance cooperation by enabling people to share information about their reasons for acting, resulting in more accurate models of one’s own and others’ behavior [67, 68].

**Fig. 2:**
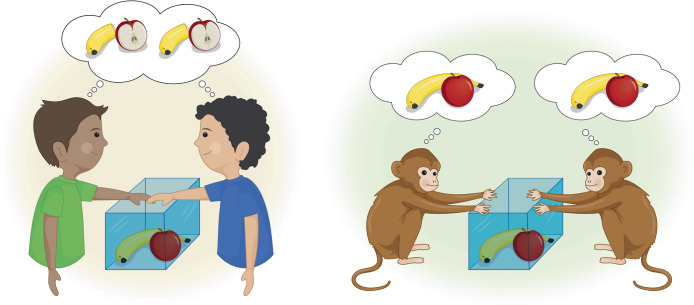
Shared intentionality in humans. Comparative and developmental work shows that humans (but not our closest primate relatives) are able to represent shared intentions and goals in a way that facilitates cooperation. Human children are more likely to work toward a common goal and share the spoils (left panel), while nonhuman primates may work together to obtain rewards but do not show evidence of commitment to shared goals (right panel).

The capacity to attribute mental states to oneself and others offers significant advantages in building shared understanding of cooperative goals and actions to achieve them. Comparative and developmental research suggests humans can represent a concept of *shared intentionality*, which brings *us* to a common understanding that *we* are jointly committed to achieving mutual goals in collaborative interactions [77] (Fig. 2). This capacity for shared intentionality motivates us to engage in cooperative acts even with distant strangers and to regulate individual desires when they conflict with collective goals. It marks a significant departure from nonhuman primates for whom the ability to think and act interdependently is thought to be much weaker, resulting in a limited ability to collaborate with joint commitment to collective goals [78].

There are several possibilities as to why humans came to exhibit this significant departure from the capacity of nonhuman primates. Firstly, it is likely to be an evolutionary adaptation to better cope with the cognitive demands from expanding social group size and complexity [12]. The demands for interdependence and collaboration over collective goals might have emerged in tandem with an increase in group size for efficient distribution of environmental resources, resulting in the development of new sociocognitive skills to address them. Another possibility is that shared intentionality may have been acquired and transmitted through social interactions more effectively in humans than our primate relatives [79]. That is, through repeated social interactions, humans might have gradually acquired the better way to collaborate for mutual benefit and passed this cumulative knowledge to later generations through cultural learning. In both cases, it is evident that shared intentionality allowed humans to become richly involved in practices of cooperative culture and norms.

Evidence for shared intentionality comes from studies comparing cooperation in human children and nonhuman primates. For example, when presented with a choice between working alone and collaborating together to obtain food, from age 2 to 3, children preferred to collaborate, while chimpanzees preferred to work alone [80]. When interrupted during a joint activity, children aged between 21 and 27 months attempted to re-engage the partner, even when the partner was not necessarily needed, suggesting that children do not consider their partner merely as instrumental to fulfilling an individual goal, but rather as collaborative partners with whom they coordinate joint intentions [81]. Moreover, 3.5-year-old children in dyadic collaborative tasks continue to collaborate until both parties obtained rewards, rather than stopping once their own reward became available, supporting their understanding of mutual commitment for all involved parties [82]. Finally, when resources were gained through collaborative effort, children shared them more equally than when resources were windfalls or resulted from individual effort; chimpanzees, meanwhile, did not show such differentiation in sharing [83]. These findings together suggest a uniquely human capacity to establish joint goals and intentions from an early age.

### Norm representation and enforcement

Shared intentionality enables humans to conceptualize *social norms:* “commonly known standards of behavior that are based on widely shared views about how individual group members ought to behave in a given situation” [84]. Research on social norms has identified a variety of different types of norms, from the mundane and conventional (e.g., norms for how to dress in different social settings) to norms that carry more moral weight (e.g., norms against lying, stealing, and cheating) [85]. Here, we focus on *cooperative norms*, which we define as social norms that facilitate cooperative interactions: i.e., norms that motivate individuals to cooperate when it is not in their economic self-interest. [84, 86, 87]

One common cooperative norm is a norm of fairness, i.e., that windfall resources should be distributed equally among group members [88]. Fairness concerns are observable across human cultures [5, 89] and emerge early in life [90]. Notably, children not only display aversion toward receiving less than others (disadvantageous inequity aversion), but also toward receiving more than others (advantageous inequity aversion) [90, 91]. Although nonhuman primates show evidence for disadvantageous inequity aversion (e.g., [92, 93]), it remains unclear if they also exhibit an aversion to advantageous inequity [94]. Other cooperative norms that appear to be unique to humans include norms of honesty, promise-keeping, and conditional cooperation [84].

When a cooperative norm is violated, humans will incur costs to enforce the norm by punishing the transgressor [95]. People are willing to enforce norms not just when they are the victim of the transgression (‘second-party punishment’), but also when they are unaffected by the transgression (‘third-party punishment’) [96, 97]. Both second- and third-party punishment contribute to the stability of cooperation [95, 96, 98]. A taste for norm enforcement emerges at a young age in humans [99]. Costly third-party punishment can be observed in children as young as 3 [100]. More broadly, from age 3, children display an awareness of normative structures governing simple rule-based games, and will intervene to teach third parties who do not follow the rules [101]. This suggests children from an early age can acquire, implement, and enforce knowledge of normative principles. In contrast, it is unclear whether nonhuman primates are willing to engage in costly norm enforcement. Experimental work demonstrates that chimpanzees engage in second-party punishment but not third-party punishment [102]. Thus, a willingness to enforce cooperative norms beyond direct retaliation may be unique to humans.

### Summary

Cooperation based on kinship and reciprocity is common to nonhuman primates and humans. However, humans are substantially more advanced in their ability to regulate individual desires towards the achievement of collective goals. Moreover, sophisticated metacognitive abilities of monitoring our own cognitive processes as well as others through mentalizing enables us to establish common understanding of joint goals and intentions. The ensuing shared intentionality, in turn, lays the foundation for uniquely human abilities to represent cooperative norms, comply with those norms oneself, and enforce norm compliance in others. These sets of advanced abilities in humans, building on one another and working in concert, may enable humans to conceptualize informal, temporally, and socially extended social contracts that play an important role in guiding cooperative behavior.

## Medial PFC and social valuation

When we make decisions, we must represent the set of available choice options, assign subjective values to the expected outcomes resulting from these options, select the most valuable option, evaluate the outcome, and update subjective value representations through learning [103]. For cooperative decisions, the computation of subjective value requires representing expected outcomes for oneself and others, and weighting those outcomes according to the anticipated responses of others and local cooperative norms [104–106]

For instance, imagine you are sitting in a park at lunchtime. Your stomach rumbles and so you begin unwrapping a sandwich you’ve just purchased at a local café. Before you can take a bite, a homeless person approaches and asks if you could spare some money for a meal. You have no cash on you. Do you offer them your sandwich?

Decisions like this engage a variety of cognitive processes. Your overall decision will depend not just on how much you value your own and others’ outcomes for their own sake, but also the relative valuation of those outcomes and the valuation of normative principles like generosity more broadly [105]. So you will need to compute the value of the sandwich not just for yourself, but also for the homeless person, how guilty you will feel if you refuse, and how virtuous you will feel if you help. These latter computations will likely depend on local cooperative norms (e.g., are you in a country where it’s expected to help strangers in need?), personal commitments (e.g., do you practice a religion which prioritizes generosity?), and whether others are watching (e.g., are you trying to impress a date?). In the next two sections, we briefly review the neural correlates of some of these computations engaged in cooperative decision-making, highlighting where relevant the neural mechanisms that appear to be common to both humans and nonhuman primates.

### Representing outcomes for self and others

Rewarding and aversive outcomes for oneself are strong motivators of decision-making. Converging evidence in nonhuman primates and humans demonstrates that motivationally salient outcomes are encoded in a valuation circuitry encompassing multiple cortical and subcortical brain regions including ventromedial prefrontal cortex (vmPFC), orbitofrontal cortex (OFC), different subregions of anterior cingulate cortex (ACC), ventral striatum, and amygdala (for comprehensive reviews, see [107–110]). These regions are active during a series of mental operations involved in value-based decision-making, from representation, valuation, and action selection to outcome evaluation and updating value representations [98, 110–111] (see Shenhav; Murray & Adolphs, this issue). In particular, studies using single-unit recordings in nonhuman primates as well as model-based fMRI in humans revealed that the neuronal/BOLD activity in these regions is parametrically modulated according to the magnitude of the positive and negative outcomes expected or received [112, 113]. Moreover, these regions respond to diverse forms of reward and punishment (e.g., money, food, pain, social approval/disapproval), providing support for a ‘common neural currency’ schema for domain-general coding of value [114].

Cooperative decisions are guided not just by outcomes for oneself, but also outcomes for others. How does the brain represent *vicarious outcomes*—i.e., motivationally salient outcomes for others? Are others’ rewards and punishments encoded in similar or distinct neural circuits to one’s own rewards and punishments? A growing body of work implicates the medial prefrontal cortex, especially the ACC gyrus, in vicarious outcome processing [115]. Initial work on this question demonstrated that the brain’s valuation circuitry responds not just to rewards for oneself, but also vicarious rewards [116–118]. For example, viewing another person winning a game evoked increased neural responses in the ventral striatum, a region in the valuation network which was also active when participants themselves experienced winning. Notably, the effect of vicarious reward in the ventral striatum was modulated by the perceived similarity to the person observed, and this modulation was indexed by increased functional connectivity between the subgenual ACC and the ventral striatum [116]. A meta-analysis of 25 neuroimaging studies investigating neural correlates of vicarious reward identified overlapping activations between personal and vicarious reward in the valuation network, including the subgenual and dorsal ACC, rostral mPFC, vmPFC, and the amygdala [119]. Vicarious reward responses in the brain are stronger in individuals higher in empathy [120] and predict cooperative behavior [121–123].

Similar evidence for vicarious neural representations in the mPFC/ACC can be found in the aversive domain. Pain delivered to both oneself and others evokes activity in the mid-ACC and anterior insula (AI), regions implicated in the affective component of pain [124, 125]. Multivariate techniques suggest that first-hand and vicarious pain experiences share common neural representations in the AI and the mid-ACC [126, 127]. Vicarious pain responses in the brain, like vicarious reward responses, track with individual differences in empathy [128] and predict cooperative behavior [129]. Empathic brain responses in the mid-ACC and AI are also highly sensitive to context, and this contextual sensitivity is thought to arise from interactions with regions implicated in mentalizing, including the dorsomedial prefrontal cortex (dmPFC) and TPJ [130–132].

Alongside studies suggesting overlapping neural representations of outcomes for oneself and others [133] there is evidence that the mPFC, and in particular the gyrus of ACC, represents distinctive information about others’ outcomes [115, 134]. Single-cell recording in nonhuman primates, which enables finer-grained examination of neuronal encoding of vicarious outcomes, suggests separable encoding of outcomes for self and others. One study demonstrated that the functionally separate but anatomically intermingled populations of neurons in the dmPFC (pre-supplemental motor area and rostral area 9) encode reward information separately for self and other [93]. In another study, where monkeys made decisions about allocating juice rewards to themselves, a conspecific monkey, both self and other, or neither, many neurons in the rostral ACC gyrus selectively encoded others’ reward outcomes, while neurons in the OFC most prominently encoded one’s own reward outcomes [135]. Rostral ACC gyrus neurons in humans also encode others’ rewarding outcomes [136]. This suggests the rostral ACC and dmPFC may be necessary for learning which actions result in positive outcomes for others, an ability crucial for the development of cooperative behavior. Supporting this prediction, lesioning the ACC (encompassing both the ventral sulcus and the gyrus) prevented monkeys from learning which actions help others [137].

### Relative and joint valuation of outcomes

Aside from representing the value of outcomes for oneself and others individually, medial prefrontal areas are also sensitive to the *relative values* of one’s own outcomes with respect to others and vice-versa. In one study, pairs of participants took part in a random drawing where one received $50 and the other received nothing. Following this, each was scanned while observing monetary transfers to self and other. For the participant who received nothing, vmPFC and ventral striatum responded more strongly to rewards for self than other. High-pay participants, meanwhile, showed the reverse pattern, with stronger responses to rewards for the disadvantaged partner than rewards for self [138]. Similarly, responses to money in the vmPFC and rostral ACC are higher when that money is offered as part of a fair split (e.g., $5 out of $10), relative to an unfair split (e.g., $5 out of $20; [139, 140]). Single-cell recordings in macaque monkeys provide convergent evidence for relative social valuation of rewards: the subjective value of rewards for self decreases in tandem with increasing reward allocations to others, and these relative values are encoded, respectively, in the dopaminergic midbrain and dmPFC [93].

In addition, there is evidence that medial prefrontal regions encode the *joint values* that arise from cooperative interactions where the whole is greater than the sum of its parts. Human fMRI studies of social exchange games like the prisoner’s dilemma show increased activity in vmPFC for cooperative relative to selfish decisions [141–143], and during repeated social interactions, activity in anterior and mid-cingulate cortex tracked the partner’s cooperative decisions [144]. Consistent with these findings, dorsal ACC neurons in macaque monkeys playing an iterative prisoner’s dilemma encoded the partner’s future decision to cooperate [145]. In the same study, a largely separate population of dorsal ACC neurons encoded the monkey’s own cooperative decisions, and disrupting dorsal ACC activity selectively inhibited mutual cooperation.

### Integrative valuation in social decision-making

Social decision-making requires not only representing the value of decision outcomes for self and others, but also integrating those values into an “all-things-considered” subjective value, or “relative chosen value” corresponding to the value of the chosen course of action relative to the alternatives. The vmPFC and dmPFC (including the dorsal ACC and pre-SMA), respectively, are suggested to positively and negatively encode relative chosen value, both for decisions that impact only oneself [107, 146–148], only others [149, 150], or both oneself and others [19, 151–154]. A recent study suggests relative value encoding in dmPFC generalizes across tasks and across self- and other-related valuations, implicating this region as a node for computing relative subjective values for self and other [150].

Making decisions on behalf of others may be particularly difficult if the other person is a stranger or if their preferences are not well understood. To overcome this uncertainty, people may engage in mentalizing processes that activate a network encompassing superior temporal sulcus, TPJ, medial precuneus, and dmPFC [155–158]. Mentalizing regions may encode subjective values themselves [154, 159] or be functionally connected with medial prefrontal valuation regions during social decision-making [149, 152, 160, 161]. Together these data support an ‘extended common currency schema’ for social decision-making, whereby social cognitive information (represented in mentalizing areas) modulates the activity of a domain-general value-representation circuitry that computes subjective value for both social and non-social decisions [105].

### Summary

Representing outcomes for oneself and others is an important first step during cooperative decision-making. Converging evidence from studies of humans and nonhuman primates demonstrates a role for medial prefrontal regions, including dorsal and ventral ACC, in encoding the value of outcomes for self and others. Medial prefrontal regions encode vicarious rewards and punishments, and individual differences in these responses predict individual differences in cooperation. These neurons are sensitive to relative and joint values in social contexts, which may explain cooperative behavior in both nonhuman primates and humans. During social decision-making, subjective values are computed in vmPFC and dmPFC, and in humans, there is evidence that these domain-general valuation regions receive input from other areas that represent social information (such as the mentalizing network). Although both humans and primates represent outcomes for self and others in medial prefrontal regions, humans go considerably further in incorporating social cognitive information into integrative values of action through actively engaging in mentalizing. In addition, human cooperative decision-making cannot be understood simply in terms of these basic outcome representations. In the next section, we’ll examine how the subjective value of cooperative decisions depends not just on relative and joint valuation of social outcomes, but also on shared beliefs about what is normatively right or wrong.

## Lateral PFC and normative behavior

When people make cooperative decisions, they consider not just the possible outcomes for themselves and others, but also the *meaning* of their actions and resulting outcomes in the context of local cooperative norms (see Glossary). As we suggested in Section 1, the ability to represent cooperative norms and use those norms to guide one’s own and others’ behavior may be unique to humans. In this section, we review work on the neural mechanisms of norm compliance and enforcement. As cooperative norms are rules that guide cooperative interactions, we begin by considering work in both nonhuman primates and humans implicating lateral PFC (lPFC; including dorsal and ventral components) in representing rules. Next, we turn to work on norm compliance and enforcement in humans, highlighting the role of lPFC and its interactions with the brain’s valuation circuitry [106, 162]. This work suggests that representations of cooperative norms in lPFC modulate the processing of outcomes for self and other in mPFC broadly and subcortical areas, enabling individuals to prioritize norm compliance and enforcement over selfish interests.

### Rule representation

The ability to represent and use rules to guide appropriate actions is a core aspect of goal-directed behavior [163–166]. Single-neuron recording studies in nonhuman primates have identified several brain regions involved in rule representation. When rhesus monkeys performed a task of switching between two rules, the activity of neurons distributed throughout the PFC, including dlPFC, vlPFC, and OFC, flexibly encoded the rule being applied [167]. Importantly, many of these neurons maintained the rule information over a sustained period of time and showed signatures of mapping the rules to a new set of stimuli, suggesting that multiple PFC areas support abstract rule representation that permits flexible applications of learned rules to new circumstances [168, 169]. Moreover, dlPFC lesions were specifically associated with the impairment in monkeys’ ability to shift between rules [169], whereas OFC lesions were selectively linked to the deficit in the capacity to reverse stimulus-reward associations, suggesting that representations of rule and reward value can be dissociated in the PFC [169].

Recent evidence shows that human lPFC computes higher-order goals based on the associations between rules and expected outcomes to ultimately guide action selection in the striatum by modulating choice-related value signals [106, 170]. More specifically, lPFC is thought to mediate a contextual modulation of subjective value by adjusting the weights assigned to multiple environmental and behavioral attributes that are integrated and mapped onto prospective outcome values (see Shenhav, this issue). This view has broadened the scope of lPFC operations to all aspects of goal-directed behavioral control. A hierarchical organization of lPFC, where contextual information propagates through the rostral-caudal gradients of abstraction, as well as its robust projections to the striatum and vmPFC further support this view [171–173]. In addition, evidence for the higher-order modulatory role of lPFC in value-based decision-making has been continuously reported in the self-control literature, establishing a potential link between lPFC and the temporal regulation of value [174–176].

Such evidence indicates that both human and nonhuman primate lPFC is centrally involved in rule representation and flexible rule-guided behavior. It is possible that humans’ exceptional ability to comply with and enforce cooperative norms may be realized by some aspects of the lPFC function that diverged between humans and nonhuman primates through evolutionary elaboration. Next, we discuss how lateral prefrontal mechanisms, in parallel with its domain general functions, contribute to norm compliance and enforcement. We note that there are no published studies of the neural basis of norm compliance and enforcement in nonhuman primates; therefore, the remainder of this section will focus on research in humans.

### Cooperative norm compliance

In line with its domain-general support for rule-guided behavior, lPFC is reliably engaged in neuroimaging studies where human participants decide whether to comply with cooperative norms. For example, in settings where fairness norms are highly salient, several studies reported increased right dlPFC activity when participants make decisions to fairly distribute money with interaction partners [177, 178]. Disrupting activity in the right dlFPC reduces the fairness of decisions in repeated interactions without affecting explicit beliefs about what is fair [179–182]. When fairness norms are less salient, dlPFC activity has been associated with less fair behavior [180, 183–185], suggesting that dlPFC may implement fairness norms in a context-specific manner.

Other work has implicated lPFC in complying with a norm of honesty, using tasks where participants are tempted to earn more money by cheating or lying. Right dlPFC and vlPFC are more active when participants respond honestly in these tasks [186–188]. Enhancing right dlPFC activity increases honest behavior [189] and patients with dlPFC lesions make fewer honest choices [190], suggesting a causal role for dlPFC in upholding norms of honesty.

Similarly, dlPFC is more active when participants uphold a norm of conditional cooperation, i.e., reciprocating others’ trusting or cooperative decisions [191–193]. Relatedly, individuals who are more prone to conditional cooperation show higher baseline tone in left dlPFC [194], and patients with damage to dlPFC are less likely to cooperate in social dilemmas [195]. Together, these findings demonstrate the engagement of lPFC regions, most commonly dlPFC, in complying with a variety of cooperative norms.

Initial theorizing on the role of lPFC in cooperative norm compliance proposed that this region implements a top-down inhibition of prepotent selfish impulses [196], drawing on classical accounts of lPFC in response inhibition [197]. More recently, lPFC’s role in norm compliance has been reinterpreted through the lens of domain-general theories of value-based decision-making [123, 180, 198, 199]. By these accounts, lPFC integrates goal-relevant information, such as norms, beliefs, and the mental states of others into value computations in a goal-directed manner [104]. Rather than inhibiting prepotent selfish responses, lPFC modulates the subjective value of behaviors that maximize selfish outcomes at the expense of norm compliance. Put simply, we do not comply with norms by overcoming a temptation to deviate, but because norm-deviant behaviors are less tempting in the first place. This mechanism is thought to operate across a variety of domains, including abstract rule-based decision-making [200] and dietary self-control [174].

LPFC modulation of subjective value is hypothesized to operate via functional interactions with valuation circuitry including vmPFC and subcortical areas [201–205]. Several studies have reported dlPFC activity when participants have to trade off benefits to oneself against cooperative norm compliance [151, 188, 206, 207]. For example, in a study where participants had the opportunity to earn money by delivering painful electric shocks to either themselves or another person, participants with stronger cooperative preferences showed decreased responses to money earned by harming others (relative to oneself) in a network of value-encoding regions including dorsal striatum and vmPFC. dlPFC tracked anticipated blameworthiness for harmful choices and showed negative functional connectivity with dorsal striatum when participants chose to forego the ill-gotten gains [19]. Another study probed the neural representation of cooperative norms by explicitly instructing participants to either focus on the ethical implications of their choices, the impact on others, or respond naturally when deciding how to allocate money between themselves and another person. Participants made more generous choices when focusing on cooperative norms and social consequences, and showed goal-sensitive encoding of choice attributes in dlPFC, such that fairness and outcomes for others were weighted more strongly when participants focused on complying with cooperative norms [208].

What motivates people to comply with cooperative norms when no one is watching? Normative and descriptive theories have highlighted a role for moral emotions like guilt in guiding compliant behavior even in the absence of external punishments. Studies using formal models of guilt aversion reveal activation in dlPFC during guilt-averse decisions [177, 191]. One study disentangling guilt and inequity aversion during a modified trust game showed that guilt aversion was associated with right dlPFC activity, while inequity aversion was reflected in the activity of the ventral striatum and amygdala, and enhancing dlPFC excitability with tDCS increased reliance on guilt-aversion [177]. When individuals can choose freely between guilt-averse and inequity-averse strategies, preference for guilt-averse strategy corresponded with multivariate patterns of activity in a network including left dlPFC, AI, mPFC, and putamen [209]. Given the relationship between guilt and anticipated blame [210], these findings dovetail with the observation of blame representation in dlPFC during cooperative decision-making [19].

### Cooperative norm enforcement

Previous theorizing has suggested norm compliance and enforcement might rely on common psychological and neural mechanisms [97, 162, 211–213]. Accordingly, lPFC (in particular dlPFC) has also been implicated in cooperative norm enforcement across diverse scenarios and tasks. Studies of norm enforcement behavior via second-party punishment in economic games show the engagement of dlPFC when participants punish others for treating them unfairly [214–216]. Disrupting right dlPFC activity with TMS reduces costly second-party punishment without affecting fairness judgments [215, 217] through functional interactions with vmPFC, which shows a reduced response during punishment decisions when right dlPFC is deactivated [215].

Norm enforcement via third-party punishment shares neural substrates with second-party punishment [218, 219]. Studies measuring both types of punishment in the same participants during economic games report common activation in dlPFC and bilateral AI [216, 220–222], findings also confirmed by meta-analysis of second- and third-party punishment studies [223, 224]. Relative to second-party punishment, third-party punishment is more likely to engage anterior vlPFC and TPJ more strongly, suggesting a greater involvement of mentalizing processes when punishing on behalf of others [224]. Consistent with these findings, patients with lesions to dlPFC and mentalizing network demonstrate atypical third-party punishment behavior [225].

During third-party punishment, dlPFC is hypothesized to integrate multiple streams of information, including the amount of harm and the intentions of the transgressor [97]. Supporting this view, in studies probing punishment decisions of criminal scenarios, dlPFC shows stronger activity for culpable acts [226] and increased functional connectivity with regions encoding mental states and harm to others [227–229]. Accordingly, disrupting activity in dlPFC with TMS interferes with the integration of information about mental states and harm to others [199]. Together these findings highlight a causal role for dlPFC in the representational integration of multiple attributes that contribute to punishment decisions.

### Summary

Cooperative norms play a central role in guiding the large-scale cooperative interactions that characterize human social life. Humans are willing to incur considerable personal costs to comply with cooperative norms and enforce those norms in others. Converging evidence implicates the lateral PFC, most commonly dlPFC, in representing cooperative norms and integrating those representations with other streams of information to guide normative behavior. Functional interactions between lateral and medial prefrontal regions suggest that the former modulates value representations in the latter in a goal-directed manner, consistent with other work highlighting a domain-general role for dlPFC in rule representation that is common to both humans and nonhuman primates.

## Future directions: anterior PFC and norm arbitration

Certain cooperative norms, such as a prohibition against physically harming an innocent person for pure personal gain, apply widely across a vast range of social and cultural settings [230]. However, other cooperative norms, such as an expectation to tip restaurant servers, are specific to particular cultures, social contexts, or social relationships. We therefore must be able to flexibly select among different cooperative norms to guide our behavior in a context-appropriate way. In this section, we identify future directions for research on the prefrontal cortex and human cooperation: how do we arbitrate between conflicting cooperative norms in situations that are ambiguous in terms of which norm to follow?

Take, for example, a set of cooperative norms around dining in a Western cultural setting. At a restaurant, a diner is expected to pay for their meal (indeed, this norm is codified into law). However, offering to pay for a home-cooked meal at a friend’s house would be seen as socially awkward or outright rude. This discrepancy can be explained by the fact that different norms operate in these different settings: the restaurant meal is governed by an (economic) *exchange norm*, where benefits are provided with the expectation of receiving a comparable benefit or payment in return, while the friend’s dinner is governed by a *communal norm*, where benefits are given without any expectation of compensation [231]. Oftentimes the relevant norm will be clear, perhaps via commonly understood “social scripts” or salient cues that indicate what is appropriate in the present situation. But what about situations where there is no clearly defined norm, such as dining out with a friend in a restaurant? Here, it may be more or less appropriate to pay for one’s own meal, depending on the size of the bill, the nature of the relationship between the friends, and the occasion of the meal—an exchange norm might be more appropriate for a business lunch between acquaintances, while a communal norm might be more appropriate for a birthday meal between a child and a parent. How do people make decisions in normatively ambiguous situations like these?

To address this question, we build on studies of how decision-makers reflect on their own choices and arbitrate between different decision strategies. This work suggests that the anterior PFC, including anterior lPFC as well as frontopolar cortex (FPC, Brodmann area 10), plays a key role in metacognition, counterfactual processing, and arbitrating between valuation systems. We suggest that *norm arbitration* might draw on similar neural processes.

Notably, FPC is unique to anthropoid primates [232], and is the largest area in human PFC. Comparative work suggests lateral FPC (lFPC) is unique to humans [233, 234], suggesting this region may support uniquely human cognition for cooperation. Moreover, existing literature supports the presence of an anterior-posterior anatomical gradient in hierarchical processing in the primate PFC, with the anterior PFC being more specialized for higher-order and metacognitive functions than the posterior PFC [11]. In this section, we first briefly review work on the neural basis of metacognition and value arbitration, and then propose how the anterior PFC (and in particular the FPC) might guide cooperative norm arbitration in humans.

### Neural basis of metacognition

Successful cooperation requires an ability to reflect on one’s own thought processes and behaviors. Numerous studies have implicated a frontoparietal network that includes FPC and anterior lPFC (alPFC; BA 47) in metacognition (for reviews see [235–238]). As described in Section 1, metacognitive abilities appear to be more extensive in humans than nonhuman primates. Nevertheless, there is some evidence that FPC, the most anterior region in the primate PFC, plays a role in monitoring decision strategies in nonhuman primates. One study found that FPC neurons retrospectively encode chosen goals as feedback approaches [239]. Such signals could be used to assess the reliability of decision strategies and monitor self-generated goals [240]. Moreover, inactivation of FPC (area 10) selectively interfered with awareness of non-experienced events, but not with experienced events, suggesting this region is causally implicated in the evaluation of one’s ignorance [241].

In humans, FPC is dramatically expanded compared with nonhuman primates [242], which may explain cross-species differences in the complexity of metacognitive processes [234, 243]. Individual variability in metacognitive accuracy (i.e., the ability to accurately judge the success of cognitive processes) is correlated with gray matter volume in mFPC [244], and patients with FPC lesions show reduced metacognitive accuracy [245], suggesting a causal role for this region in metacognitive performance. During value-based decision-making (in a task where hungry participants chose between different snack foods), alPFC encoded subjective confidence in choices, and showed functional connectivity with value-encoding regions of vmPFC. This functional connectivity, in turn, predicted individual variability in the relationship between confidence and choice accuracy [246].

One important aspect of evaluating the quality of one’s decisions is keeping track of counterfactual outcomes: is the grass greener on the other side of the fence? Multiple studies have shown that FPC prospectively tracks counterfactual evidence, including the reward value of unchosen options [247] and counterfactual prediction errors [248]. When choices lead to unexpected outcomes, alPFC activity mediates the impact of postdecision evidence on choice confidence, suggesting it may guide changing one’s mind on the basis of new evidence [249].

There is also evidence that FPC prospectively tracks internal variables that bear on the future success of decisions. During perceptual decision-making, people prospectively estimate an internal probability of making a correct choice, and these estimations are tracked in mFPC and alPFC [250]. A similar process may take place during self-regulation by precommitment. When people limit their access to tempting small immediate rewards, lFPC is more active, and connectivity between lFPC and the frontoparietal control network is stronger in people who stand to benefit more from precommitment [47]. A subsequent study showed that enhancing lFPC activity with anodal transcranial direct current stimulation selectively increased decisions to precommit [251]. These findings suggest that FPC and alPFC may orchestrate self-regulation in part through accessing internal signals that convey the likely success of different decision strategies.

Together these studies suggest that FPC, in particular its more lateral aspects that underwent a dramatic expansion over the course of human evolution, plays a fundamental role in enabling people to evaluate the quality of their decisions both prospectively and retrospectively—an important aspect of adjusting cooperative behavior across social contexts. However, it is important to note that the neural evidence of metacognition is not limited to the FPC. In fact, multiple regions in the FPC network, encompassing ACC subregions and mPFC subareas, will likely synergistically contribute to the metacognitive process. For instance, confidence-related signals have been observed in the perigenual ACC [252, 253] and dmPFC [253] for making decisions relevant for self and other. Moreover, the highly integral functions of ACC in flexibly implementing behaviors should play an important role in the FPC network with respect to metacognition and other cognitive processes underlying cooperative decision-making (see Monosov & Rushworth, this issue).

### Arbitrating between valuation systems

Research on the neurocomputational mechanisms of value-based learning and decision-making reveals that there are multiple valuation systems in the brain that employ different algorithms for learning the expected value of actions and outcomes. In particular, an important distinction has been made between ‘goal-directed’ and ‘habitual’ systems, which are proposed to rely on model-based and model-free reinforcement learning algorithms, respectively [254, 255]. The computationally expensive model-based algorithm learns contingencies between actions and outcomes to build a model of the world, and selects actions by prospectively searching through the model and selecting a course of action that serves current goals. Meanwhile, the computationally efficient model-free algorithm assigns values to actions through trial and error, and habitually selects actions with the highest cached value. Oftentimes the goal-directed and habitual systems agree on what is the best choice, but sometimes they give conflicting answers.

Recent work has examined the neurocomputational mechanisms supporting arbitration between model-based and model-free control over decision-making. An early computational account suggested an uncertainty-based arbitration, whereby control is exerted by the system with the lowest uncertainty in its value predictions [256]. Building on this account, the “mixture of experts” framework proposes that the brain monitors the reliabilities of the predictions of different valuation systems (the ‘experts’), and uses those reliabilities to allocate control over behavior [257] (see O’Doherty & Averbeck, this issue). This arbitration mechanism, proposed to rely on anterior PFC (including FPC and vlPFC), assigns a weight to each expert on the basis of its reliability, gating the extent to which that expert’s recommended policy contributes to action selection, and transmits this information to the vmPFC, which serves as the system’s output channel, encoding an integrated subjective value. The final policy arises from combining across the opinions of the individual experts, weighted by their relative confidence. Therefore, rather than implementing only one strategy at a time dictated by the dominating expert system, the brain can efficiently and flexibly utilize collective expertise of different systems. This model bears some resemblance to the optimal integration model of sensory perception, whereby the combination of multiple sensory cues is achieved by linear summation of population activity generated by each sensory cue [258–260]. In both models, the final policy (or percept) is influenced more strongly by the more confident “voice”.

Evidence for the mixture of experts model comes from fMRI studies of human subjects in learning environments where different learning strategies are variably successful over time. In a study of arbitration between model-based and model-free systems, reliabilities for both systems were encoded in vlPFC and FPC [261]. Enhancing vlPFC activity with anodal tDCS increased model-based control, while inhibiting vlPFC activity with cathodal tDCS had the opposite effect, suggesting vlPFC gates the extent of default model-free control over behavior, amplifying model-based control when advantageous [262]. Another study showed that vlPFC tracks the reliabilities of emulative and imitative strategies during observational learning, with imitative strategy employed as a default [263]. Finally, a study of arbitration between individual experience and social advice also revealed reliability signals of each strategy encoded in right FPC and vlPFC [264]. Together these findings provide initial support for a domain-general arbitration process in anterior PFC that polls the reliabilities of different learning and decision-making systems to allocate control over behavior.

### Arbitrating between cooperative norms

We suggest that a process similar to the mixture of experts model is likely to guide arbitration between different cooperative norms in guiding context-appropriate social behavior. That is, norm arbitration might involve allocating control over behavior by weighting the reliabilities of value predictions generated by different cooperative norms. We propose that anterior PFC computes the reliabilities of the expected values of different behavioral policies prescribed by any cooperative norms being considered. These predictions may be generated based on the presence of contextual cues that indicate whether a particular norm is relevant for the current context. The posterior over the behavioral policies can therefore be obtained as a weighted sum over the predicted values of cues indicating different norms, producing graded levels of confidence over the chosen policy.

**Fig. 3:**
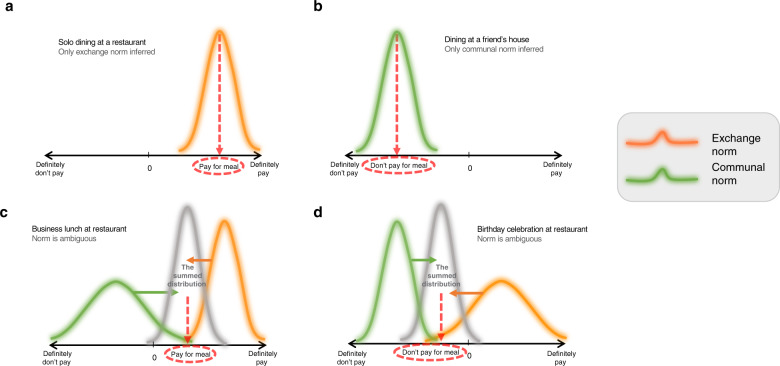
Cue-guided cooperative norm arbitration. Graphical depiction of arbitration between communal and exchange norms across various social settings. **a** When dining alone at a restaurant, salient cues indicating an exchange norm is appropriate result in a more confident prediction about the expected value of paying for one’s meal. **b** When dining at a friend’s house, salient cues indicating a communal norm is appropriate result in a more confident prediction about the expected value of not paying for one’s meal. **c**, **d** When you are having a business lunch or having a birthday celebration at a restaurant, there is no obvious norm. Therefore, cues indicating communal or exchange norms increase the precision of the respective predictions. The downstream decoder in anterior PFC allocates weights over two norms based on their relative reliabilities, which are determined as a function of each norm’s expected value and (inverse of) uncertainty [273]. Two weighted distributions are linearly summed to evaluate the confidence in choosing the policy of communal norm over exchange norm, based on a mechanism informed by the multi-sensory cue integration literature [274]. This way, the decisions are adjusted flexibly based on the linear summation of the relative reliability of two norms. Even when the decision is expressed as binary choice between two presented options, because the final decision is made on the basis of weighted average, the relative reliability of the relevant norms alter the degree of confidence about the decision.

Consider the example that opened this section: how do you decide whether to pay for your meal when dining? There are two potentially relevant cooperative norms that make opposite behavior policy recommendations: an exchange norm that dictates you should pay for your meal, and a communal norm that suggests you should not. When you are dining solo at a restaurant (Fig. 3a), the exchange norm is strongly confident in its recommendation that paying your bill is the best option because there are only exchange cues present; when invited for dinner at a friend’s house, the communal norm makes a robust prediction that you should avoid pulling out your wallet when dessert is served, because there are only communal cues present (Fig. 3b). But what should you do when dining at a restaurant with a friend (Fig. 3c, d)? This will depend on the relative numbers and strengths of surrounding communal and exchange cues. For instance, if you and your friend have met in the cafeteria of your office building and have brought some work materials to discuss (an exchange cue), the exchange norm will make a more reliable prediction and you will feel more confident about asking for separate checks (Fig. 3c). Whereas if you are celebrating your friend’s birthday and have brought them a birthday card (a communal cue), the communal norm will make a more reliable prediction and you will feel more confident about treating them to their meal (Fig. 3d).

Empirical evidence for this hypothesis is so far scarce. One possible explanation for this missing evidence is that most studies of social decision-making in humans consider only a single social context or norm, applied over a very brief timescale. For instance, neuroeconomic paradigms typically consider how individuals implement fairness norms in one-shot interactions. Such paradigms generally do not require participants to arbitrate between behavioral policies, but instead to consider implementing a single policy (such as a norm of fairness or reciprocity).

However, preliminary support for norm arbitration in FPC comes from neuroimaging studies of social decision-making that require participants to consider multiple decision contexts and strategies. One early study examined the neural basis of compliance with a fairness norm in two distinctive contexts: one where punishment was possible, and another where it was not. The contrast between the two conditions revealed increased activation in alPFC and vlPFC as well as dlPFC [178]. Another study measured the brain activity of individuals playing a repeated public goods game where they faced a series of decisions about whether to contribute some money to benefit their entire group. In this setting, individuals must trade off selfish concerns against long-term group benefits. Because participants interacted repeatedly within the same group, it was possible to dissociate brain signals encoding how much an individual stood to benefit from the current decision (individual utility) and how much the group could benefit from the remaining interactions (group utility). While individual utility was encoded in the vmPFC, group utility was encoded in the lFPC, and changes in individual choice strategies were mediated by functional interactions between lFPC, dorsal ACC, and vlPFC [265]. lFPC has also been implicated in adaptively choosing how to publicly communicate private mental states. In a task where participants privately assess their decision confidence and adapt their confidence reports to different social partners in order to maximize rewards, signals in lFPC tracked with social contexts requiring higher adjustments of confidence reports, and multivariate activity patterns in lFPC represented distinguished between these social contexts [266]. Together these findings converge on a role for anterior PFC in adaptively adjusting decision-making across different social contexts.

Finally, the anterior PFC undergoes a protracted period of development, rapidly increasing its volume and dendritic complexity throughout late childhood and adolescence [267–269] (see Kolk & Rakic, this issue). Intriguingly, developmental studies also show that children increasingly adjust their moral judgments to social-relational context as they get older. For example, while older children (aged 6–7) and adults believe that authority figures are more obligated than ordinary individuals to punish wrongdoing, but ordinary individuals are not, younger children (aged 4–5) believe that everyone is obligated to punish [270]. Relatedly, older children and adults believe that friends are more obligated to help one another than strangers, while younger children believe that everyone is equally obligated to help one another [271]. Whether the increasing sensitivity of moral judgments to relational context depends on the development of anterior PFC is an intriguing question for future study.

Of course, there may be other possible mechanisms that support arbitration between different cooperative norms. One alternative possibility is a “winner-take-all” mechanism whereby detection of a cue signaling one norm over another prompts a categorical dominance of the relevant norm, rather than the weighted averaging approach described above. Such a mechanism would be akin to sensory cue-separation where one of two channels dominates, rather than aggregating across multiple channels as in sensory cue integration [258, 259, 272]. Another possibility is that certain cooperative norms, like a prohibition against physically harming others, are so deeply ingrained and apply so universally that they dominate across contexts operating more like a Pavlovian reflex or habit [17, 273, 274]. Future work could fruitfully adjudicate between these possibilities.

### Summary

The anterior PFC, in particular the FPC, has dramatically expanded over the course of human evolution and is implicated in a variety of cognitive processes that may be unique to humans, including advanced metacognitive abilities, the capacity to represent and learn from complex counterfactuals, and arbitrating between different strategies for learning and decision-making. Building on this work, we propose that the anterior PFC supports norm arbitration: determining which cooperative norm(s) to apply in a particular social context. Preliminary evidence for this hypothesis comes from neuroimaging studies of adaptive social decision-making, which show that the anterior PFC (including anterior vlPFC and lFPC) encodes variables that are necessary for maximizing rewards across diverse social contexts. We further hypothesize that there is a possible anterior-posterior gradient of norm processing such that norm arbitration is carried out by more anterior aspects of the PFC, whereas norm representation is mediated by more posterior aspects of the PFC. Future work can extend these findings by probing the engagement of anterior PFC in tasks where participants must use cooperative norms to guide decisions across different cultural or relational contexts.

## Conclusion

The prefrontal cortex, in particular its more anterior regions, has expanded dramatically over the course of human evolution. In tandem, the scale and scope of human cooperation has dramatically outpaced its counterparts in nonhuman primate species, manifesting as complex systems of moral codes that guide normative behaviors even in the absence of punishment or repeated interactions. Here, we provided a selective review of the neural basis of human cooperation, taking a comparative approach to identify the brain systems and social behaviors that are thought to be unique to humans. Humans and nonhuman primates alike cooperate on the basis of kinship and reciprocity, but humans are unique in their abilities to represent shared goals and self-regulate to comply with and enforce cooperative norms on a broad scale. We highlight three prefrontal networks that contribute to cooperative behavior in humans: a medial prefrontal network, common to humans and nonhuman primates, that values outcomes for self and others; a lateral prefrontal network that guides cooperative goal pursuit by modulating value representations in the context of local norms; and an anterior prefrontal network that we propose serves uniquely human abilities to reflect on one’s own behavior, commit to shared social contracts, and arbitrate between cooperative norms across diverse social contexts. We suggest future avenues for investigating cooperative norm arbitration and how it is implemented in prefrontal networks.

## Glossary

Cooperation: any behavior that is potentially costly to an individual but benefits at least one other individual [8].
Self-regulation: adjusting one’s inner states or behaviors according to personal goals, expectations or standards [34].
Metacognition: ability to monitor, assess and orchestrate one’s own cognitive processes and their quality for the guidance of behavior[48, 50, 53, 275].
Precommitment: voluntary restriction of access to temptations on the basis of a metacognitive insight that one’s own self-regulation is likely to fail [40–46].
Mentalizing: capacity to infer and represent mental states (i.e., desires, intentions and beliefs) of oneself and others and thereby better predict and regulate future behaviors [276].
Shared intentionality: ability to build a common understanding of joint commitment to a collective goal with others to engage in cooperative acts and to regulate individual desires when they conflict with the collective interest [277].
Social norm: commonly known rules or standards of behavior that are based on widely shared views about how individual group members ought to behave in a given situation [84].
Cooperative norm: a type of social norm that facilitates cooperation [84, 86, 87].
Norm compliance: adoption of behaviors constrained by normative considerations: pursuing prescribed behaviors and avoiding proscribed behaviors according to social norms [198].
Norm enforcement: inducing others to obey a social norm through sanction in the event of norm violations [86].
Norm arbitration: flexible selection of norms to guide one’s behavior in a context-appropriate way, especially when the situation is ambiguous and no predominant norm is inferred.
